# Genomic Insights Into Five Strains of *Lactobacillus plantarum* With Biotechnological Potential Isolated From *chicha*, a Traditional Maize-Based Fermented Beverage From Northwestern Argentina

**DOI:** 10.3389/fmicb.2019.02232

**Published:** 2019-09-25

**Authors:** Lidia Rodrigo-Torres, Alba Yépez, Rosa Aznar, David R. Arahal

**Affiliations:** ^1^Departamento de Microbiología y Ecología, Universitat de València, Valencia, Spain; ^2^Colección Española de Cultivos Tipo (CECT), Universitat de València, Valencia, Spain; ^3^Instituto de Agroquímica y Tecnología de los Alimentos, Consejo Superior de Investigaciones Científicas, Valencia, Spain

**Keywords:** *Lactobacillus plantarum*, folates, riboflavin, food safety, genome analysis

## Abstract

Lactic acid bacteria (LAB) are indigenous microorganisms that have been involved in food fermentations throughout history to preserve food and also to give special characteristics to them. The traditional fermented foods that are still being elaborated in indigenous populations around the world are a potential source of LAB with important biotechnological properties and/or beneficial to health. In a previous work, LAB biodiversity associated with *chicha*, a traditional maize-based fermented beverage from Northwestern Argentina, was studied, both by culture dependent and independent methods. From that study, 392 isolates were recovered, mostly members of *Lactobacillus* and *Leuconostoc*. Biotechnological characterization of representative isolates led to the selection of five strains belonging to the species *Lactobacillus plantarum* for their ability to produce vitamin B2 (riboflavin) and vitamin B9 (folates), their antimicrobial properties and antibiotics susceptibility. In this work, we present the Whole Genome Sequences (WGS) of these five strains that have been deposited in the Spanish Type Culture Collection: M5MA1 (= CECT 8962), M9MM1 (= CECT 8963), M9MM4 (= CECT 8964), M9MG6 (= CECT 8965), and M9Y2 (= CECT 8966), and a detailed description of their characterization, through a genomic approach, analyzing the genes responsible for these biotechnological properties, making a comparative study of the five genomes and reporting the aspects related to food safety, in accordance with the recommendations of the European Food Safety Authority (EFSA FEEDAP Panel, 2018) aiming at their use in the design of functional foods. The analysis unveiled, for the five strains, the complete set of genes for folate and riboflavin biosynthesis, the absence of pathogenic factors, the presence of CRISPR and *cas* genes, phage sequences, insertion elements and an aminoglycosides resistance gene, *aadA*, whose resistance could not be proved phenotypically in any strain. Genomic comparisons showed that strain CECT 8962 was significantly different in terms of genetic content and allowed the identification of carbohydrates metabolism and membrane transport related genes as the main components of the unique and accessory genome.

## Introduction

Lactic acid bacteria (LAB) are naturally present in a wide number of food materials, fermented foods, and beverages (Stiles and Holzapfel, 1997; Steinkraus, 2002; Tamang et al., 2016b). Many of them, typical of indigenous cultures, are still elaborated in different countries and regions of the world, frequently in isolated communities, and maintained as a home production through artisanal processes (Tamang et al., 2016b; Jiménez et al., 2018; Väkeväinen et al., 2018). Cereal fermented products, in particular those derived from maize, are very important in Latin America and have been consumed as main staple food for centuries. Among them, *chicha* is the most important traditional fermented beverage, which is produced since pre-Hispanic times in northwestern regions of Argentina, and Andean regions of Bolivia, Colombia, Ecuador, and Peru, being mainly consumed by the native population during religious and agricultural festivities as well as during family and social events (Lorence-Quiñones et al., 1999; Delibes and Barragan, 2008).

It is well known that naturally fermented foods and beverages contain both functional and non-functional microorganisms (Tamang et al., 2016a). Recently, many studies focused in the selection of microorganisms associated with fermented foods and beverages, a few of them including *chicha*, that show functional properties such as antimicrobial activities, nutrient synthesis, degradation of anti-nutritive compounds such as phytates, or production of enzymes (Tamang et al., 2009; Bourdichon et al., 2012; Jung et al., 2013; Thapa and Tamang, 2015; Yépez et al., 2017). Among the natural microbial populations, LAB display a high biosynthetic capacity and therefore, during fermentation, some of them may utilize raw materials leading to the production of exopolysaccharides, vitamins, bioactive peptides, etc. to enrich the nutritional value of some fermented foods. As a result, they will confer health benefits to the consumers, as well as contribute in food safety by inhibiting the proliferation of other undesirable microorganisms (Magnusson et al., 2003; Farhad et al., 2010). For this reason, LAB have been used in food processing with both technological and functional purposes (Axelsson et al., 2012; Holzapfel and Wood, 2014).

In fact, many *Lactobacillus* species have a long history of human usage (Bernardeau et al., 2006), including the recognition as Generally Recognized as Safe or a Qualified Presumption of Safety by the Food and Drug Administration (FDA) and European Food Safety Authority (EFSA), respectively (Leuschner et al., 2010). In human nutrition, only the species used as starter cultures are considered food ingredients in the European Union regulation. However, food safety is also required when microorganisms are present in the food (Laulund et al., 2017). With this regard, EFSA has developed a guidance which establishes the characterization of microorganisms in terms of taxonomic identification, sensitivity to antibiotics, production of antimicrobials, production of toxic metabolites (i.e., biogenic amines), toxicity and pathogenicity, and proposes the analysis of the genome as an unequivocal diagnostic tool (EFSA FEEDAP Panel, 2018).

Advances in genome sequencing technologies as well as the growing availability of tools for sequence analysis have nowadays expanded the use of whole genome analysis in microbiology. In addition to taxonomic or phylogenetic studies, it is being applied for food safety assessment of strains (EFSA FEEDAP Panel, 2018) as well as to expand their biotechnology potential through comparative genomics (Sun et al., 2015).

In previous studies, our research group combined high-throughput sequencing (HTS) technologies and culture-dependent methods to study the LAB biodiversity associated with *chicha* (Elizaquível et al., 2015) and performed the characterization of their biotechnological potential (Yépez et al., 2017). Out of 392 LAB isolates screened, five *Lactobacillus plantarum* were selected for their antimicrobial activity against foodborne bacterial pathogens and spoiler fungi, B-vitamin production, i.e., B2 (riboflavin) and B9 (folates) and phytate-degrading activity, being besides sensitive to antibiotics (EFSA FEEDAP Panel, 2012). In addition, *L. plantarum* is extremely well-adapted to different niches due to a highly flexible genome with life-style islands mainly related to the utilization of carbohydrates (Kleerebezem et al., 2003) which points to these strains as good candidates for functional food design among other food applications.

The first *L. plantarum* genome was described in 2003 (Kleerebezem et al., 2003) and the second, 6 years later, included a recommendation, for all strains to be used as probiotic, to undergo complete genome sequencing (Zhang et al., 2009). Here we present the Whole Genome Sequence (WGS) of the five *L. plantarum* strains isolated from *chicha*, the taxonomic identification and analysis of their biotechnological properties by genetic exploration of the genomes of each strain individually and comparatively. Besides, an analysis of the factors related to food safety was also carried out through a genomic approach, in accordance with the EFSA recommendations (EFSA FEEDAP Panel, 2018).

## Materials and Methods

### Bacterial Strains

Strains M5MA1, M9MM1, M9MM4, M9MG6, and M9Y2 were isolated in 2011 from *chicha*, a maize-based fermented beverage produced in Maimará (Jujuy), Argentina (Elizaquível et al., 2015). MRS agar and MRS broth were used as routine cultivation media and incubations were done at 30°C, 48 h. They were deposited at CECT with numbers CECT 8962, CECT 8963, CECT 8964, CECT 8965, and CECT 8966, respectively.

### Vitamins Production and Phytate-Degrading Activity

The production of folates and riboflavin was evaluated following the methods already described (Laiño et al., 2013; Juarez del Valle et al., 2014) and based on microbiological bioassays. In the case of phytate-degrading activity, it was tested using the procedure described by Anastasio et al. (2010).

### Whole Genome Sequencing and Metabolic Pathways Analysis

Genomic DNA was isolated using Real Pure Spin kit (Durviz) following the standard protocol recommended by the manufacturer. The integrity of the extracted DNA was checked by visualization in a 2.0% (w/v) agarose gel with 0.5 × Tris-borate-EDTA (TBE) electrophoresis at 80 V, 1 h (as recommended by the sequencing service). Its purity and quantity were checked by measuring the absorbance at 260 and 280 nm with a spectrophotometer Nanodrop2000c (Thermo Fisher Scientific) and calculating the ratio A260/A280. Genome sequencing was achieved at Central Service of Support to Experimental Research (SCSIE) of the University of Valencia (Valencia, Spain) using an Illumina Miseq technology with 2 × 250 paired-end reads.

The Illumina reads were analyzed for quality control using FASTQC, a common quality control tool developed by Babraham Bioinformatics to check raw sequencing data. After filtering, the remaining reads were assembled using the software Spades 3.9.0 (Nurk et al., 2013). A plot, coverage versus length of the contigs, was performed to help in the choice of the parameters for contigs filtering. After the filtration of contigs [500 base pair (bp) length and 10–50 × kmer coverage], evaluation of the final assembly against a reference genome was done with the software QUAST v4.3 (Gurevich et al., 2013). The bioinformatic tool CheckM v1.0.7 (Parks et al., 2015) was used to assess the genome quality prior to annotation using Prokka v1.12 (Seemann, 2014) and RAST v2.0 (Rapid Annotation using Subsystem Technology) (Aziz et al., 2008). The process of quality assessment of reads, read-processing, assembly and annotation with Prokka was carried out in Linux OS, other tools were accessed online. BlastKOALA^1^ and Kyoto Encyclopedia of Genes and Genomes (KEGG) Mapper (Kanehisa et al., 2018) were used to annotate the genomes using KEGG Orthology (KO) and to analyze metabolic pathways. Reference genes of vitamins biosynthesis pathways were extracted from KEGG Gene Database^2^ when necessary, and BLASTp^3^ was used to align those genes to the genomes of the *L. plantarum* strains considered in this study.

Tools in RAST and KEGG servers were utilized together to corroborate and strengthen the search for the genes involved in the metabolic pathways of vitamin synthesis. In RAST, genes involved in the synthesis of folates and riboflavin were searched by going through “Browse annotated genome in SEED viewer,” functional category of “Cofactors, vitamins and prosthetic groups” and “Folate biosynthesis” and “Riboflavin biosynthesis” subsystems. In the case that routes were not complete, but considering the positive capacity in the laboratory, further investigation was carried out using the KEGG tools described as follows: genomes were annotated by assigning KO numbers with BlastKOALA and metabolic pathways were reconstructed by “Reconstruct module” which renders: (i) metabolic pathways named “Modules”; (ii) metabolic pathway completeness or incompleteness, i.e,. if one or two enzymatic blocks are missed, it is specified in each module; if more than 2 blocks are missed, the module is indicated as “incomplete.” When one or more enzymatic blocks were missed the gene encoding for the corresponding enzyme was searched in the KEGG Genes database looking for a reference gene of the same species; and then they were blasted to the genomes under study through the BLASTp tool. If no significant results were found with this workflow, the search was considered finished and the absence of that gene in the genome was assumed.

### Strains Identification: 16S rRNA Gene Sequence and Genomic Relatedness Indexes

For identification purposes, the entire sequence of the 16S rRNA gene, extracted from the annotated genomes, was compared to EzBioCloud database (Yoon et al., 2017), a curated and comprehensive resource of prokaryotic type strains. When that comparison was >98.6%, the similarity between genomes was assessed using the index Average Nucleotide Identity with BLAST (ANIb) algorithm in JSpeciesWS (Richter et al., 2016).

### Genomic Comparisons

Genomic comparisons of the five strains of *L. plantarum* were performed by the “Compare” and “Sequenced based” tools of SEED Viewer in RAST server v2.0 (Aziz et al., 2008). The tool only allows the comparison of four genomes against a reference. The output is a table that includes the genes of the reference organism in chromosomal order and the hits on the compared organisms accordingly. Also, the result is represented in a graphical circular representation of the genomes showing colored genes according to the protein sequence identity (%) and if they are bidirectional or unidirectional best hits.

The pangenome, core-genome and COG and KEGG distribution of genes were also investigated with the program BPGA v1.3 (Chaudhari et al., 2016). In BPGA an identity cut-off of 50% similarity is used to make orthology clusters of genes among genomes in the first step and clusters with at least one gene per genome are taken to define the core genome. COG and KEGG assignments are made on the basis of best hits with the respective database.

### Safety Assessment

Genomes were screened with tools recommended in the EFSA Guidance (EFSA FEEDAP Panel, 2018) to investigate about relevant genes involved in food safety. Resistome prediction was assessed by Resistance Gene Identifier (RGI) tool of the Comprehensive Antibiotic Resistance Database (CARD)^4^ (Jia B. et al., 2017) with two approaches (i) using contigs file with the parameters “Perfect and strict hits only” and “High quality/coverage”; (ii) using the annotated (proteins) file with the parameters “Perfect and strict hits only” and “Low coverage” to improve matching in the search. PathogenFinder^5^ (Cosentino et al., 2013) was used for prediction of bacterial pathogenicity with the model of prediction “All” representing the whole-data model (model that performed best with the phylum *Firmicutes* according to authors), with contigs and proteins fasta files as input. BAGEL4 webserver^6^ (Van Heel et al., 2013) was utilized to predict genes coding for bacteriocins and ribosomically synthetized and post-translationally modified peptides (RiPPs). Metabolic prediction of biogenic amines biosynthesis was conducted with BLASTp alignment of reference genes involved in synthesis of histidine, tyramine, putrescine (Table 1) against protein fasta files of studied genomes. Genome stability was investigated by analyzing: (i) prophages with PHASTER webserver^7^ (Arndt et al., 2016); (ii) the presence of clustered palindromic interspaced palindromic repeats (CRISPR) regions and *cas* genes with CRISPRCasFinder^8^ (Couvin et al., 2018), using contigs fasta file as input, selecting “Perform cas detection” and leaving the rest of parameters by default; (iii) insertion sequences (IS) with ISfinder^9^ (Siguier et al., 2006), using contigs fasta file as input file and BLASTn tool with default parameters; and (iv) plasmids by two methods: PlasmidFinder^10^ and manual search of plasmid genes.

**TABLE 1:** Reference genes for the biosynthesis of histamine, tyramine, and putrescine.

**Biogenic amines**	**Biosynthesis pathway**	**NCBI accession**	**Strain**
Histamine	Histidine decarboxylase (*hdcA*) pathway	LN877767	*L. reuteri* IPLA11078
Tyramine	Tyrosine decarboxylase (*tyrRS*) pathway	NC_008497.1, gene ID: 4413406, 4413405, 4413407	*L. brevis* ATCC 367
Putrescine	Ornithine decarboxylase (*odc*) pathway	KT020759	*L. rossiae* D87
Putrescine	Agmatine deiminase (*aguA*) pathway	AF446085.5	*L. brevis* IOEB 9809

## Results and Discussion

### Genome Sequence Metrics and Relatedness Indexes

Assembly parameters, genome length, % G + C molar content, protein, rRNA and tRNA genes and accession numbers in public databases (EMBL/Genbank/DDBJ) are summarized in Table 2. It is noteworthy that the majority of genomes drafted in this study have more than 100 contigs and a N50 below 100,000 bp despite of having an assembly coverage higher or equal 100× and more than 250× depth of coverage. These values could reflect the complexity of the genomes in terms of repeat regions or mobile elements. Genome complexity often affects *de novo* assembly of prokaryote genomes especially when using the short reads generated by the most common sequencers (Schmid et al., 2018).

**TABLE 2:** General features of the genomes utilized in this study.

**Strain**	**N° Contigs**	**Genome size (Mb)**	**N50 (bp)**	**Depth of coverage**	**Assembly coverage**	**Completeness**	**Contamination**	**G + C (mol%)**	**Protein genes**	**rRNA genes**	**tRNA genes**	**Accession**
*L. plantarum* CECT 8962	136	3.3	103,668	272×	197×	100	0.52	44.3	3103	7	63	OKQP01^∗^
*L. plantarum* CECT 8963	136	3.2	54,053	326×	239×	99.83	0.52	44.4	3055	8	57	OKQT01^∗^
*L. plantarum* CECT 8964	173	3.2	48,177	248×	79×	99.60	0.00	44.4	3031	9	60	OKQV01^∗^
*L. plantarum* CECT 8965	152	3.3	54,128	241×	179×	99.83	0.52	44.3	3144	8	59	OMOO01^∗^
*L. plantarum* CECT 8966	161	3.3	54,053	325×	251×	99.46	0.00	44.3	3094	9	61	OMOP01^∗^
*L. fabifermentans* DSM 21115^T^	199	3.3	29,340	NA	100×	99.07	1.39	45.0	2992	4	60	AYGX02
*L. herbarum* TCF032-E4^T^	132	2.9	36,760	NA	413×	99.02	3.09	43.4	2636	4	50	LFEE01
*L. paraplantarum* DSM 10667^T^	258	3.4	93,771	NA	100×	99.38	3.09	43.7	3096	11	50	AZEO01
*L. pentosus* DSM 20314^T^	152	3.6	48,077	NA	100×	97.22	2.16	46.3	3122	7	66	AZCU01
*L. plantarum* subsp. *argentoratensis* DSM 16365^T^	230	3.2	54,882	NA	100×	99.07	2.78	45.0	2851	5	67	AZFR01
*L. plantarum* subsp. *plantarum* ATCC 14917^T^	36	3.2	152,365	NA	36×	99.38	2.78	44.5	2960	2	60	ACGZ02
*L. xiangfangensis* LMG 26013^T^	122	3.0	77,513	NA	100×	99.02	2.47	45.1	2682	5	55	JQCL01

**^∗^This study; NA, not available.**

Complete 16S rRNA gene sequence extracted from genomes were 1,565 bp in length in all five strains. These sequences showed a similarity of 99.93% to *L. pentosus* DSM 20314^T^ followed by 99.87% to *L. paraplantarum* DSM 10667^T^ and *L. plantarum* subsp. *plantarum* ATCC 14917^T^, and 99.80% to *L. plantarum* subsp. *argentoratensis* DSM 16365^T^. To precise the identification, ANIb was calculated among genomes of closely related type strains (>98.6% of 16S rRNA gene sequence similarity) and the resulting values are included in Table 3. Thus, all strains belong to *L. plantarum* species, which is included in the QPS list of EFSA (EFSA Scientific Committee, 2007).

**TABLE 3:** Similarity matrix showing the ANIb values between the strains under study (belonging to *L. plantarum*) and type strains of related species (16S rRNA gene sequence similarity >98.6% in EzBioCloud Database).

	**ANIb**	**1**	**2**	**3**	**4**	**5**	**6**	**7**	**8**	**9**	**10**	**11**	**12**
1	*L. plantarum* CECT 8962	–											
2	*L. plantarum* CECT 8963	**98.4**	-										
3	*L. plantarum* CECT 8964	**98.4**	**100.0**	–									
4	*L. plantarum* CECT 8965	**98.3**	**99.8**	**99.8**	–								
5	*L. plantarum* CECT 8966	**98.3**	**99.8**	**99.8**	**100.0**	–							
6	*L. fabifermentans* DSM 21115^T^	75.3	75.3	75.3	75.3	75.3	–						
7	*L. herbarum* TCF032-E4^T^	76.7	76.7	76.7	76.7	76.8	74.5	–					
8	*L. paraplantarum* DSM 10667^T^	85.6	85.5	85.5	85.6	85.6	74.9	77.1	–				
9	*L. pentosus* DSM 20314^T^	79.4	79.4	79.4	79.4	79.5	75.0	76.5	79.4	–			
10	*L. plantarum* subsp. *argentoratensis* DSM 16365^T^	94.9	95.0	95.0	95.0	95.0	74.9	76.6	85.4	79.8	–		
11	*L. plantarum* subsp. *plantarum* ATCC 14917^T^	**98.5**	**98.6**	**98.6**	**98.6**	**98.6**	74.5	76.7	85.2	79.2	94.9	–	
12	*L. xiangfangensis* LMG 26013^T^	76.2	76.2	76.2	76.3	76.2	75.4	75.7	76.1	79.8	76.3	75.4	–

**Values above the threshold for species delineation (95%) are in bold.**

### Biotechnological Traits: Vitamins Production and Phytate-Degrading Activity

**FIGURE 1:**
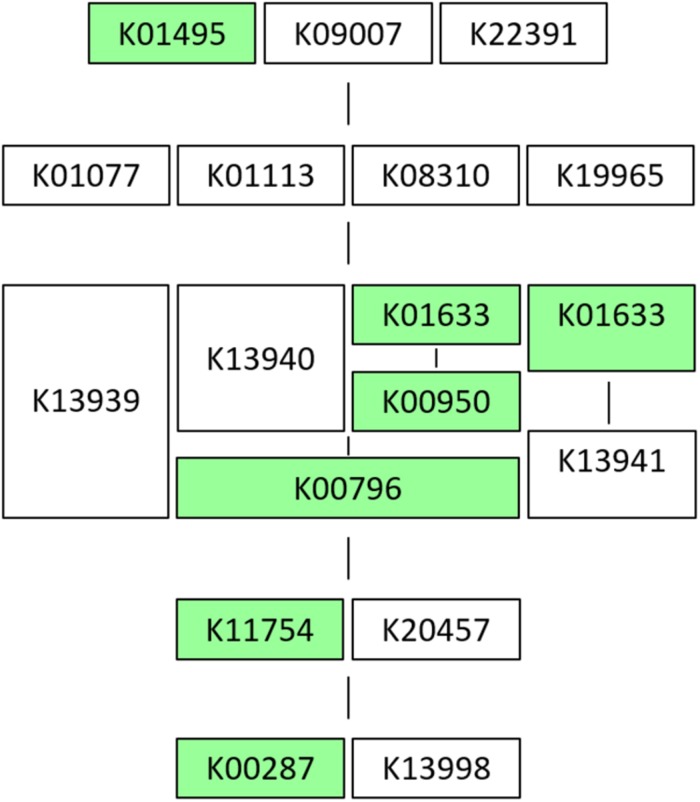
KEGG scheme in strain CECT 8962 of M00126: Tetrahydrofolate biosynthesis from GTP. The module is defined as follows (logic definition): (K01495, K09007, K22391) [K01077, K01113 (K08310, K19965)] [K13939, (K13940, K01633, K00950) (K00796), (K01633, K13941)] (K11754, K20457) (K00287, K13998), where the genes are named with K number based on the KEGG Orthology, parenthesis indicate an enzymatic block, commas, “OR,” space or “+,” mean “AND” (for a molecular complex). Scheme of the pathway: green charts mark genes that are present in the genome submitted. For this strain there is one enzymatic block missing [K01077, K01113 (K08310, K19965)]. K01077 = E3.1.3.1, *phoA*, *phoB*; alkaline phosphatase [EC:3.1.3.1], K01113 = *phoD*; alkaline phosphatase D [EC:3.1.3.1], K08310 = *nudB*, *ntpA*; dihydroneopterin triphosphate diphosphatase [EC:3.6.1.67], K19965 = *folQ*; dihydroneopterin triphosphate pyrophosphohydrolase [EC:3.6.1.67].

Results on the *in vitro* vitamin production tests revealed folates biosynthesis by the five *L. plantarum* strains in concentrations between 30 and 55 ng/mL (Table 4). These are moderate values compared to those reported by Carrizo et al. (2016) for strains recovered from quinoa sourdough (16–143 ng/mL). However, when genomes were analyzed in RAST, the “Folates biosynthesis” subsystem showed that all of them lacked the *folQ* gene coding for dihydropterin triphosphate pyrophosphohydrolase (EC:3.6.1.67), the second enzyme acting in the pathway for transformation of GTP to tetrahydrofolate (THF). Folates incomplete pathway were also revealed by KEGG tools, which pointed again to the same missing enzymatic block in all strains (Figure 1). In order to assess this contradictory result, the gene lpl_3295, coding for dihydropterin triphosphate pyrophosphorylase (YP_004890815.1), from the genome of *L. plantarum* WCFS1, was used as reference and blasted against the genomes under study. Then, a high similarity match (query cover equal or bigger than 98% of amino acid sequence, 97% of identity and *e*-value smaller or equal to 10^–136^) was obtained with the gene encoding a dITP/XTP pyrophosphatase in all genomes. This gene has no KO assigned which explains why *folQ* gene was not found by BlastKOALA. Thus, the ability to produce folates was corroborated by the presence of the complete pathway for THF biosynthesis in all strains (Tables 4, 5).

**TABLE 4:** Vitamins production and phytate-degrading activity.

**Strain**	**Riboflavin production (ng/mL)**	**Folates production (ng/mL)**	**Phytate-degrading specific activity (U/mL)**
CECT 8962	158.61	54.66	66.21
CECT 8963	109.17	51.96	379.63
CECT 8964	160.08	41.63	186.16
CECT 8965	139.29	32.43	188.06
CECT 8966	122.92	40.37	154.36

**TABLE 5:** Presence of genes involved in folates (B9 vitamin) and riboflavin (B2 vitamin) biosynthesis pathways.

**Strain**	**Genes involved in folates synthesis**							**Genes involved in riboflavin synthesis**					
		
	***folA***	***folB***	***folC***	***folE***	***folK***	***folP***	***folQ***	***ribBA***	***ribD***	***ribE***	***ribH***	***yitU***	***ribF***
CECT 8962	×	×	×,×	×	×	×	×	×	×	×	×	×	×,×
CECT 8963	×	×	×,×	×	×	×	×	×	×	×	×	×	×,×
CECT 8964	×	×	×,×	×	×	×	×	×	×	×	×	×	×,×
CECT 8965	×	×	×,×	×	×	×	×	×	×	×	×	×	×,×
CECT 8966	×	×	×,×	×	×	×	×	×	×	×	×	×	×,×

**×, a single copy of the gene; ×, ×, two copies of the gene.**

Regarding the riboflavin *in vitro* production all studied strains were able to produce the vitamin in concentrations above 100 ng/mL, being these natural production values similar, or even higher, to those described by other authors in the literature (Capozzi et al., 2011; Juarez del Valle et al., 2014). Genomic analysis of the subsystem “Riboflavin, FMN, FAD metabolism” in RAST server showed that all strains harbored the genes involved in the pathway (*ribBA*, *ribD*, *ribE*, *ribH*, *yitU*, *ribF*) and position of these genes was correlative which confirms their location in the same operon. Moreover, KEGG tools subscribed that module M00125 of riboflavin biosynthesis from GTP was complete (Table 5).

Phytate-degrading activity was observed in the five strains ranging from low (66.21 U/mL) to high values (379.63 U/mL). This activity has been described in LAB strains from sourdough (Anastasio et al., 2010) or quinoa (Carrizo et al., 2016) sources reaching significant levels around 600 and 710 U/mL, respectively. Previous studies demonstrated that phytate-degrading activity of Lactobacilli can be efficiently carried out by non-specific acid phosphatases (Haros et al., 2008).

### Safety Assessment

Although *L. plantarum* is a species with QPS status, aiming to the use of the strains under study in food applications, their genomes were evaluated to cover all the safety concerns as recommended for food additives containing live microorganisms (Zhang et al., 2012). Therefore, following recommendations of EFSA Guidance for characterization of microorganisms used as food additives in animal feed and as producing organisms (EFSA FEEDAP Panel, 2018) the genome analysis consisted of: taxonomic identification, antimicrobial resistance (AMR) to antibiotics categorized as Critically Important Antimicrobials (CIA) and/or Highly Important Antimicrobials (HIA) by the World Health Organization^11^, virulence and pathogenicity determinants and antimicrobials production.

#### Identification

Identification of strains employing genetic and genomic methods, as mentioned above, confirmed that all strains belong to the species *L. plantarum* (Table 3).

#### Antimicrobial Resistance

RGI tool predicted the gene *aadA*, for inactivation of aminoglycosides, in the five genomes. In all cases, the prediction was marked as “perfect hit” or “strict hit” according to RGI criteria (Table 6) using the “protein homolog prediction model” which detects a protein sequence based on its similarity to a curated reference sequence. This model, as described in CARD, determines the strength of a match based on one parameter: a curated BLASTp bitscore cutoff of 450. Matches are categorized as “perfect,” “strict” or “loose.” A perfect match is 100% identical to the reference sequence along its entire length; a strict match is not identical but the bitscore of the matched sequence is greater than the curated BLASTp bitscore cutoff; and a loose match is outside the cut-off value to provide detection of new emergent threats or more distant homologs of AMR genes, but can also render spurious hits.

**TABLE 6:** AMR gene search results by the RGI tool of the CARD Database (using contigs file as input) and AMR *in vitro* assay results.

**Strain**	**RGI criteria: AMR gene**	**Reference gene in CARD database**	**Reference Cover (%) (amino acid sequence)**	**Identity (%) (amino acid sequence)**	**Bit score**	**AMR (*in vitro* assay results)^∗^**	**Genes in the vicinity of *aad*A gene in Prokka/RAST**
*L. plantarum* CECT 8962	Perfect hit: *aadA*	WP_001206316.1	127.46	100	523.1	S	RNAi/no gene
*L. plantarum* CECT 8963	Strict hit: *aadA2*	AAF27727.1	129.73	86.72	454.5	S	RNAi/hypothetical protein
*L. plantarum* CECT 8964	Strict hit: *aadA15*	ABD58917.1	59.70	100	147.1	Amp	Only predicted by RGI-CARD
	Strict hit: *aadA21*	AAN87151.1	76.81	99.5	407.5		RNAi/no gene
*L. plantarum* CECT 8965	Perfect hit: *aadA*	WP_001206316.1	127.76	100	523.1	S	No gene/hypothetical protein
*L. plantarum* CECT 8966	Strict hit: *aadA15*	ABD58917.1	57.79	100	151.8	Amp	Only predicted by RGI-CARD

**^∗^Yépez et al. (2017); S, sensitive to all antibiotics tested; Amp, Ampicillin resistance.**

Some differences were found between the analysis using contigs file or proteins file as input data in RGI and between the programs and databases utilized for the prediction. When using contigs file, RGI performs both annotation and BLASTp comparison against the CARD database. When using proteins file, it only makes BLASTp comparison of the proteins given directly to the CARD database. This was reflected in the results of the analysis using contigs as input: “perfect hits” were retrieved from strains CECT 8962 and CECT 8965, with *aadA* as the best hit ARO (Antimicrobial Resistance Ontology) term, with 523.1 as bit score; but using proteins as input, “strict hit” with *aadA2* was obtained as the best hit ARO term, with 455.3 of bit score, in both strains (data not shown). Differences in the accuracy was also noted between CARD, Prokka and RAST regarding AMR gene prediction: gene *aadA15* was predicted in strains *L. plantarum* CECT 8964 and CECT 8966 only with RGI of CARD, while RAST and Prokka did not predict it (Table 6). That could be due to the low coverage of the query gene sequence (<60%, in both cases) with the reference gene which is reflected in the low bit score value (Table 6). Our results highlight the need to combine both sequence analysis tools and databases to assess findings derived from genome analysis approaches.

Aminoglycoside antibiotics affect protein biosynthesis acting over the 30S ribosomal subunit and they are primarily used in the treatment of infections caused by gram-negative aerobic bacilli, staphylococci, and other gram-positive and against gram-negative in combination with beta-lactams exerting a synergic effect (Krause et al., 2016). Their action requires respiration; therefore, anaerobic bacteria are intrinsically resistant to them. The *aadA* genes encode ANT enzymes, one of the three types of aminoglycoside modifying enzymes [acetyltransferases (AAC), nucleotydiltransferases (ANT) and phosphotransferases (APH)]. ANT enzymes mediate specifically the resistance against spectinomycin and streptomycin. The *aadA* genes are organized in cassettes which can be found as gene fusions being part of plasmids, integrons and transposons (Ramirez and Tolmasky, 2010).

Antimicrobial resistance of the five strains under study had been assayed previously *in vitro* against ampicillin, chloramphenicol, clindamycin, erythromycin, gentamicin, kanamycin, streptomycin, tetracycline and vancomycin (Yépez et al., 2017). Strains *L. plantarum* CECT 8964 and CECT 8966 showed resistance to ampicillin (Table 6), but none of them showed resistance to gentamicin, kanamycin or streptomycin, the three aminoglycosides tested, based on the EFSA cut-off values.

**FIGURE 2:**
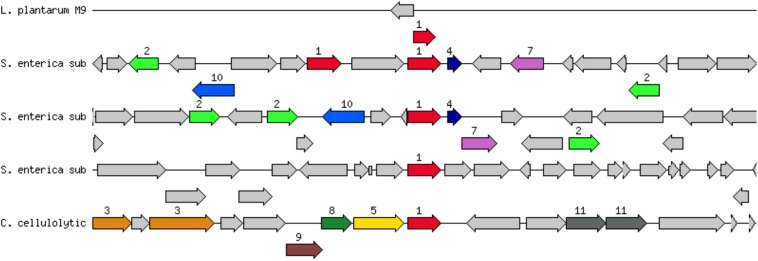
Genetic context of *aadA2* gene (streptomycin 3″-O-adenylyltransferase (EC 2.7.7.47) @ Spectinomycin 9-O-adenylyltransferase) in *L. plantarum* CECT 8965 (*L. plantarum* M9, first line in the image). The *aadA2* gene (red arrow, immediately below the line) is located alone with a hypothetical protein (gray arrow, over the line) in the holder contig. Closely related genes and their context are shown below: S. enterica sub = *Salmonella enterica* subsp. *enterica* serovar Choleraesuis strain SC-B67, C. cellylolytic = *Clostridium cellulolyticum* H10. Description of genes in the closest strain of *Salmonella enterica* subsp. *enterica* serovar Choleraesuis strain SC-B67, from left to right: resolvase, unknown function, 2: mobile element protein, transposon Tn21 modulator protein, 10: integron integrase Intl1, hydrolase, alpha/beta fold family, Phosphoserine phosphatase (EC 3.1.3.3), 1: streptomycin 3″-O-adenylyltransferase (EC 2.7.7.47), florfenicol/chloramphenicol transporter FloR, 1: streptomycin 3″-O-adenylyltransferase (EC 2.7.7.47) @ Spectinomycin 9-O-adenylyltransferase, 4: ethidium bromide-methyl viologen resistance protein EmrE, transposase, 7: dihydropteroate synthase (EC 2.5.1.15), unknown protein, dehydrogenases with different specificities (related to short-chain alcohol dehydrogenases), macrolide-efflux protein, 2: mobile element protein, unknown function, unknown function, ADP-heptose:LPS heptosyltransferase.

EFSA Guidance 2018 states that when Minimal Inhibitory Concentration (MIC) is smaller or equal than the cut-off values stablished, but AMR genes related with that susceptibility are present, the likelihood of the AMR gene to become active should be evaluated (EFSA FEEDAP Panel, 2018). Therefore, a deeper analysis on the sequence and genomic context was addressed to predict activity and/or mobility of *aadA* gene in the strains. About sequence identity, the gene *aadA* of strains CECT 8962, CECT 8963, CECT 8964 and CECT 8965 showed a gene coverage >70% and had identity >85% against reference genes in CARD database (Table 6). Genomic context was also analyzed to get more information about the probability of activation and to inquiry about horizontal gene transfer (HGT). Surprisingly the *aadA* gene was located alone in the holder contig, or only with an additional gene, in all strains. The genome annotation by Prokka unveiled a RNAi next to the *aadA* gene in strains CECT 8962, CECT 8963, and CECT 8964. However, annotation by RAST did not predict any gene next to *aadA* in the holder contig in strains CECT 8962 and CECT 8964, and only a hypothetical protein in CECT 8963 (Figure 2). Inactivation of the *aadA* gene by RNAi would explain why these strains were sensitive to the aminoglycosides tested. There were no genes annotated by Prokka next to the *aadA* gene in strains CECT 8965 and CECT 8966 but RAST predicted a hypothetical protein in strain CECT 8965. Thus, no conclusion could be made about the probability to become active or about transferability in these strains. The nature of the *aadA* gene, usually linked to mobile elements, and attending that breakpoints in *de novo* assembly occurs often in repetitive regions could explain the location of this gene in a single contig (Treangen and Salzberg, 2011).

No perfect or strict hit was predicted by RGI with genes involved in ampicillin resistance in strains CECT 8964 and CECT 8966, thus according to the guidance, no further studies are required and these strains do not pose a risk on ampicillin resistance transfer.

#### Virulence or Pathogenicity

Analysis of pathogenicity factors using contigs or proteins as input files exhibited the same results: none of the strains contained pathogenicity factors. In all cases, the probability of being pathogen was low (<0.21 out of 1) and all the strains were predicted as non-human pathogens. These results were consistent with the QPS status of *L. plantarum*.

#### Production of Antimicrobials

**FIGURE 3:**
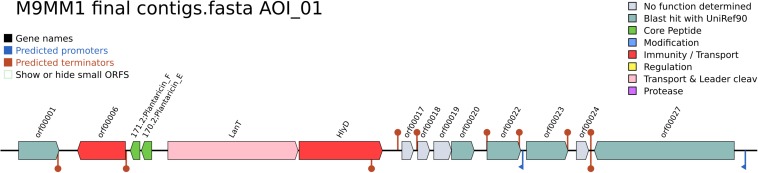
Area of Interest (AOI) of *L. plantarum* CECT 8963 in contig 8. Genes with function determined from left to right (gene name, function and locus tag): orf00001, response regulator PlnD (LAP8963_00903, LAP8963_03147); orf00006, P71468_LACPL PlnI (Immunity protein PlnI, membrane-bound protease CAAX family) (LAP8963_00904); 171.2 Plantaricin_F, plantaricin F (LAP8963_00905); 170.2 Plantaricin_E, plantaricin E (LAP8963_00906); LanT, bacteriocin ABC-transporter, ATP-binding and permease protein PlnG (LAP8963_00908); HlyD, accessory factor for ABC-transporter PlnH (LAP8963_00906); orf00017, function not determined, orf00018, function not determined, orf00019 function not determined, orf00020, putative membrane peptidase YdiL (LAP8963_00911), orf00022, UPF0177 protein YxdF (LAP8963_00912); orf00023, PlnS (LAP8963_00913); orf00024, function not determined, orf00027, DNA helicase IV (LAP8963_00915, LAP8963_00763).

Antimicrobial activity against foodborne pathogens and spoiler fungi was screened previously for all the *L. plantarum* strains (Yépez et al., 2017). All of them were capable of inhibiting at least two of the four spoiler fungi tested and the three pathogenic bacterial strains. Cell free supernatants (CFS) exerted antibacterial and antifungal activity against at least one fungal strain, and, in some cases the bacterial inhibition proved to rely on substances of proteinaceous nature. In this case, to continue the study of antimicrobial compounds production, genome mining was performed with BAGEL4 webserver. All strains, except *L. plantarum* CECT 8962, displayed three bacteriocins clusters, named as “Areas of Interest” (AOI): *L. plantarum* CECT 8963 had AOI of (size in bp): 12,300 (contig 8) (Figure 3), 3,343 (contig 92), 3,021 (contig 94); strain CECT 8964, 5,187 (contig 86), 3,341 (contig 98), 3,090 (contig 100); strain CECT 8965, 12,300 (contig 9), 3,343 (contig 99), 3,021 (contig 102); and strain CECT 8966, 12,300 (contig 9), 3,412 (contig 94), 3,090 (contig 99). The three AOIs consisted of plantaricin E/F, plantaricin J, and plantaricin A as core proteins, respectively, in all strains. These findings were in line with the proteinaceous nature of antibacterial substances in CFS of strains CECT 8963 and CECT 8965. Regarding EFSA recommendations, no special considerations apply to these strains as they are members of a QPS species and they are not aimed to be used as producer microorganisms.

#### Biogenic Amine Genes

Biogenic amines are nitrogenous compounds usually originated from decarboxylation of amino acids and can cause toxicological effects if large amounts accumulate in food. Fish and fermented food are the most common food containing biogenic amines, specially histamine, tyramine and the polyamine putrescine, and LAB can be responsible for their production (Spano et al., 2010). Genes encoding biogenic amines were screened by BLASTp against reference genes of histamine biosynthesis via histidine decarboxylase, tyramine via tyrosine decarboxylase, putrescine via agmatine deiminase and ornithine decarboxylase (Table 1). Criteria for determining a positive result were *e* < 10^–3^, 70% of query cover and 30% identity of amino acid sequences (Salvetti et al., 2016). The results were negative in all cases, with the exception of genes *hisS* (histidyl-tRNA synthetase) and tyrosine-tRNA ligase, which are housekeeping genes Therefore, based on genomic data, the use of these strains in food fermentation do not potentially pose a risk regarding production of biogenic amines.

#### Genome Stability

**FIGURE 4:**
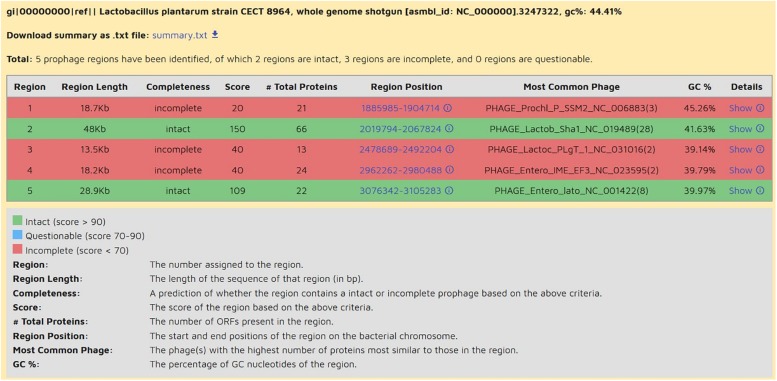
List of prophage regions of *L. plantarum* CECT 8964, as shown in Summary tab of PHASTER webserver. Regions are classified into three categories indicated with colors depending on completeness: intact (green), blue (questionable), and red (incomplete). Strain CECT 8964 displays 2 intact prophage regions (green) and 3 incomplete regions (red). Region 2 is in contig 27 and region 5 is composed of five contigs 81, 82, 83, 84, 85.

Genomic stability is an important factor to be determined in microorganisms destined for food applications due to the possible horizontal transfer of pathogenic or AMR genes to the gut microbiota. Thus, presence of prophages, plasmids and insertion elements should be investigated. Prophage regions were analyzed with PHASTER webserver. Genome accession numbers were introduced as input. The results delivered by the program are shown in three tabs: Summary, Details and Genome viewer. In Summary tab, putative regions are displayed and signaled by colors (Figure 4) and named as “intact,” “questionable,” and “incomplete” depending of phage sequence completeness. All strains investigated enclosed intact regions for at least one phage (Table 7). Additionally, strains CECT 8964, CECT 8965 and CECT 8966 contained “incomplete” regions, and CECT 8963, CECT 8965, and CECT 8966, “questionable” regions. As PHASTER program concatenates the contigs to perform the analysis, then, as a second step, “intact” regions were blasted against genomes to detect the contigs that harbor these prophage regions. As a result, it was seen that in almost all cases, the prophagic regions were distributed among several contigs (Table 7). Considering that the complex nature of prophage regions hinders the *de novo* genomic assembly, this result is not surprising. Presence of prophage in the genomes entails the possibility of mobilization of genetic factors, and thus, it is important to determinate if that regions contain pathogenic or AMR genes. On the other hand, prophages are genetic elements frequently present in *L. plantarum* strains (Ventura et al., 2003; Briggiler Marcó et al., 2012; Liu et al., 2015), and are a problematic issue in milk industry sometimes yielding low-quality products (Garneau and Moineau, 2011). Further analysis will be necessary to study the lysogenic capacity of these prophages and foresee their activation in the products where the carrier strain will be inoculated.

**TABLE 7:** Contigs matching prophage regions (intact) in each genome and predominant phage sequences predicted in each region.

**Prophage regions**	**Kb**	**Match (BLASTn)**	**Predominant phage**
*L. plantarum* CECT 8962			
Region 1	67.4	Contig 4	*Gordonia* phage Zirinka
Region 2	52.5	Contig 9	*Lactobacillus* phage Sha1
*L. plantarum* CECT 8963			
Region 2	50.4	Contig 29, 30	*Lactobacillus* phage Sha1
Region 4	31.5	Contig 79, 80, 81, 82, 83, 84	Coliphage phi-X174
*L. plantarum* CECT 8964			
Region 2	48	Contig 27	*Lactobacillus* phage Sha1
Region 5	28.9	Contigs 81, 82, 83, 84, 85	Coliphage phi-X17
*L. plantarum* CECT 8965			
Region 2	45.8	Contigs 27, 28	*Lactobacillus* phage Sha1
Region 4	52.6	Contigs 79, 80, 81, 82, 83, 84, 85, 86	Coliphage phi-X17
*L. plantarum* CECT 8966			
Region 3	50.4	Contigs 27, 28	*Lactobacillus* phage Sha1

Immunity against phages can be achieved with the presence of CRISPR and the collaborative endonucleases Cas (Mojica et al., 2005). CRISPR are repeat arrays displayed in the DNA of many bacteria and archaea. The repeats range from 23 to 47 bp, show palindromic (or almost palindromic) symmetry, and are separated by spacers of similar length, usually with a unique sequence in the genome, that match phage, plasmid and mobile elements sequences. CRISPR-associated genes (*cas*) are located in the vicinity of CRISPR sequences and play different roles, recognizing foreign DNA and incorporating spacers into DNA, maturation of CRISPR transcript RNA (crRNA) and defensive recognition and interference with the foreign DNA. The screening of CRISPR sequences by CRISPRcasFinder webserver resulted in at least two CRISPR regions in each genome but in all cases only one region had *cas* genes nearby. For the type of *cas* genes, these CRISPR regions are of class 2 type II, which provide immunity against foreign DNA (and not RNA) (Makarova et al., 2015). CRISPR regions without *cas* genes are thought to be non-functional (Zhang and Ye, 2017). Therefore, CRISPR-cas system may prevent these *L. plantarum* strains to acquire AMR or pathogenic genes through HGT (Marraffini and Sontheimer, 2008).

ISfinder unveiled IS in almost all contigs in all genomes. Most of them belonged to the genus *Lactobacillus*: *L. plantarum* (ISLpl1, ISLpl2, ISLpl3, ISP1, ISP2), *L. sakei* (ISLsa1, IS1163), *L. sanfranciscensis* (IS153), *L. helveticus* (ISLhe4, ISLhe30); or *Pediococcus pentosaceus* (ISLpp1) but also from other Gram-positive bacteria, *Enterococcus faecium* (ISEfa9) and *Enterococcus hirae* (IS1310); *Bacillus thuringiensis* (IS240F); *Desulfitobacterium hafniense* (ISDha13); and Gram-negative bacteria, *Escherichia coli* (Tn2), *Salmonella enterica* (Tn3). These findings supported the complex nature of the *L. plantarum* strains of this study.

Regarding plasmid content in the *L. plantarum* strains, the *de novo* assembly of 250 bp paired reads did not allow to achieve the completion of these genomes as entire chromosome/s and plasmid/s. However, plasmids are known to be present in many *L. plantarum* (Van Kranenburg et al., 2005; Heiss et al., 2015) and are of concern in terms of HGT of pathogenic and AMR genes. Plasmids where screened by PlasmidFinder which could not find any plasmid sequence. Only manual search succeeded in finding plasmid genes along RAST and Prokka annotation files. Analysis on the RAST annotation file released *parA* and *parB* genes encoding the NTPase motor-protein and centromere-binding protein for type I segregation system of plasmids, respectively, in the five genomes of *L. plantarum*. Particularly, two *parB* genes were found in all genomes, while *parA* genes were present in different number: six in CECT 8962, five in CECT 8965 and CECT 8966; four in CECT 8963 and three in CECT 8964. Besides, all genomes harbored an additional putative partition protein which matched with *parA* genes using BLASTn tool. As *parAB* genes are located as a single copy in plasmids (Guynet and De la Cruz, 2011), this may indicate the presence of at least two plasmids in all strains. In contrast, only one plasmid initiation replication protein gene (*rep*) was predicted in all strains. *Lactobacillus* plasmids are usually replicated with the rolling-circle method (Jalilsood et al., 2014) and thus *rep* gene is an essential gene involved in plasmid replication. Therefore, without completion of the draft genomes, it is difficult to precise the number of plasmids in each strain, but multiple other genes (conjugation protein, integrase/recombinase plasmid associated, LacX protein, plasmid, site-specific recombinase, DNA invertase Pin related protein) also supported that plasmids are present in the studied *L. plantarum* strains.

### Genomic Comparisons

Genomic comparisons were conducted to enlighten common, accessory and unique factors among *L. plantarum* CECT 8962, CECT 8963, CECT 8964, CECT 8965, and CECT 8966. Seed Viewer browser of RAST server was employed to make sequence-based comparisons, with *L. plantarum* CECT 8962 as the reference genome because of its outstanding characteristics (Yépez et al., 2017). 240 unique genes (0% protein similarity) were found only in this strain, the majority of them hypothetical proteins and prophage genes (Table 8), and 146 low-similarity genes (0 < % protein similarity ≤50). This singularity was supported by previous studies that revealed best performance regarding behavior under technological stress conditions, such as high and low temperatures, high salt concentrations, low pH and different ethanol concentrations (Yépez et al., 2019). Accordingly, genome analysis showed the presence of genes containing conserved domains that can be identified as putatively involved in general stress responses, such as universal stress proteins UspA, osmotic stress, heat shock (HtpX proteins), oxidative stress (including a methionine sulfoxide reductase B), also described by Jia F.-F. et al. (2017) for *L. plantarum*, as well as CspA and CspC, highly homologous cold-shock proteins already characterized by Kleerebezem et al. (2003).

**TABLE 8:** Number and function of unique genes in *L. plant*arum CECT 8962 when compared to strains CECT 8963, CECT 8964, CECT 8965, CECT 8966 (RAST results).

**Number of genes**	**Encoded enzyme or gene name**
1	2-amino-3-carboxymuconate-6-semialdehyde decarboxylase (EC 4.1.1.45)
1	ABC-type nitrate/sulfonate/bicarbonate transport systems, Periplasmic components
1	Cell surface protein precursor
1	DUF1706 domain-containing protein
1	Ferrichrome-binding periplasmic protein precursor (TC 3.A.1.14.3)
1	FtsK-like DNA segregation ATPase, YDCQ *B. subtilis* ortholog
1	Glucarate dehydratase (EC 4.2.1.40)
1	NADH pyrophosphatase (EC 3.6.1.22)
1	O-acetyltransferase
1	Oxidoreductase (putative)(EC:1.-)
1	Permease of the major facilitator superfamily
1	Type III restriction-modification system methylation subunit (EC 2.1.1.72)
1	Stage V sporulation protein K
1	Imidazolonepropionase related amidohydrolase
1	TolA protein
1	Pullulanase (EC 3.2.1.41)
1	ThiJ/PfpI family protein
1	Prephenate dehydratase (EC 4.2.1.51)
1	Putative MR-MLE-family protein
1	Putative MDR permease; possible multidrug efflux pump
1	Replication protein
2	ADP-ribosylglycohydrolase
2	Flavodoxin (1), Putative flavodoxin (1)
2	Glycosyltransferase
2	Putative dienelactone hydrolase and related enzymes
4	Integral membrane protein (2), Putative membrane protein (1), Membrane protein involved in the export of O-antigen, teichoic acid lipoteichoic acids (1)
3	Transcriptional regulators
4	Transposase (1), Transposase, fragment (3)
4	Unknown
5	Mobile element
5	Polysaccharide biosynthesis protein (putative) (3), Capsular polysaccharide synthesis enzyme Cap8 (1), PTS system, cellobiose-specific IIC component (EC 2.7.1.69) (1)
6	Type I restriction-modification system proteins
40	Phage genes
140	Hypothetical protein

**240**	

Even though all five *L. plantarum* strains display stress response machinery, special proteins such as nitrate/sulfonate/bicarbonate ABC transporter were only identified in *L. plantarum* CECT 8962. This system has been also described in a comparative study of *L. plantarum* genomes from potential probiotic strains by Li et al. (2016) and it is up-regulated in response to salt-stress, increasing the efficiency of inorganic salt uptake and giving the bacteria a way to survive and compete under osmotic stress conditions.

The role of LAB in the biotransformation of carbohydrates is relevant for different biotechnological applications, such as the transformation of raw materials, the optimization of bacterial growth and the production of valuable metabolites. In the comparative genomic study of 213 lactobacilli carried out by Sun et al. (2015), 22 out of 95 families of glycosyltransferases (GT) recorded in CAZy database^12^ were found, which showed a high level of GT-encoding diversity. According to this high variability, in the present study, two exclusive genes encoding for a GT were found in *L. plantarum* CECT 8962. Another unique gene found in this strain is the one codifying for the cellobiose-specific IIC component in the phosphoenolpyruvate-dependent sugar phosphotransferase system (PTS). The PTS is a major carbohydrate active-transport system in bacteria that catalyzes the phosphorylation of sugar substrates to cross the microbial cell membrane. Kwak et al. (2016) detected specific genes for this component in a genome comparative analysis in *L. plantarum* GB-LP2, referring this fact to an evolutionarily accelerated adaptation of this strain to vegetable-based fermentation.

**FIGURE 5:**
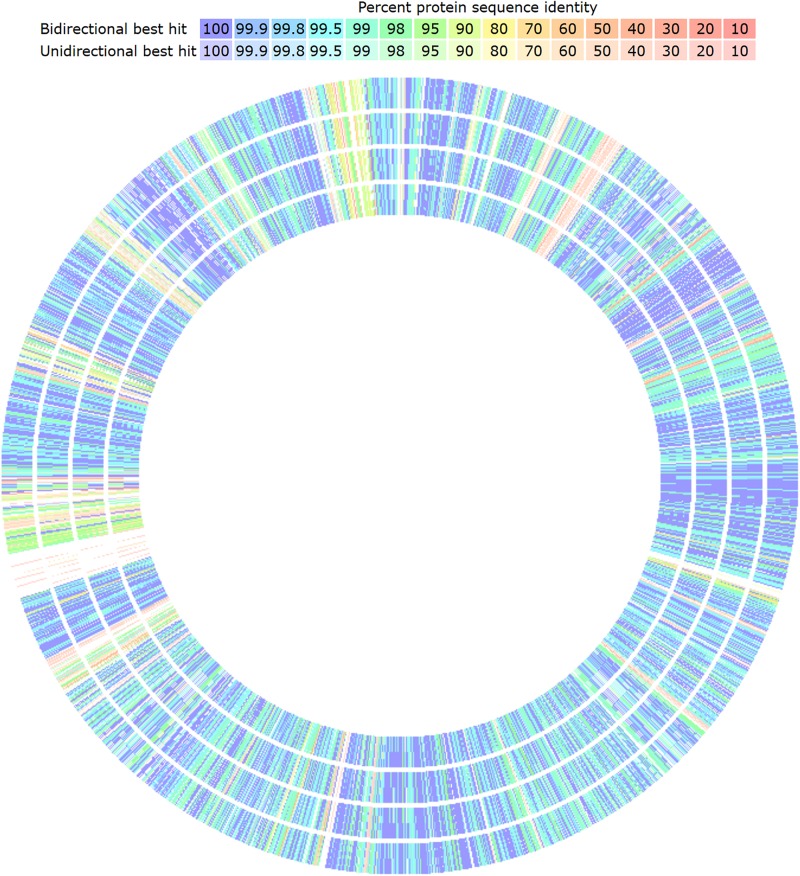
Graphical genomic comparison map of *L. plantarum* strains with Seed Viewer sequenced-based comparison tool in RAST server. *L. plantarum* CECT 8962 was taken as reference (not shown). From outer to inner ring: strain CECT 8965, CECT 8963, CECT 8964, and 8966. Colors denote amino acid similarity percentage to the reference genome, from purple (100%) to light red (10%).

The genomic comparison of strains, having CECT 8962 as reference, revealed several low or non-similarity regions among strains (Figure 5). These regions contained mainly genes belonging to the mobilome as mobile elements, phage proteins, transposases but also *par* genes and *trs* genes of the conjugative gene transfer complex, that could point to the possibility of HGT through plasmid conjugation (Grohmann et al., 2003). The most important particular facts of strain CECT 8962 are the large number of hypothetical proteins (140 out of 240 exclusive genes) (Table 8), which would lead to further investigation about singular functions attributed to this strain. Carbohydrate transport and metabolism related genes were remarkably outnumbered in these regions with low similarity. Some genes coding for PTS transport system proteins, glycosyl transferases, and two pullulanases (starch hydrolytic enzymes) were encountered as exclusive for strain CECT 8962 and could confer advantages in the adaptation to amyllaceous matrices as well as potential industrial applications (Hii et al., 2012). Polysaccharide biosynthesis and export coding genes were enclosed with exclusivity in this strain, and among them, proteins related to export of O-antigen and teichoic and lipoteichoic acids. Sortases, internalin-like, mucus-binding proteins and the operon *opp* were also present uniquely in strain CECT 8962. Sortases are enzymes that modify cell surface proteins including pilins, enzymes and glicoproteins and are related to adhesion to host cells and pathogenesis (Spirig et al., 2011). Additionally, internalin-like proteins can attach E-cadherin in epithelial cells as was reported by Sabet et al. (2005). *Opp* operon codifies for an ATP−binding cassette−type transporter associated with membrane proteins involved in the uptake of oligopeptides, but has been related to other physiological functions, e.g., adhesion, biofilm formation, and cell wall recycling (Nepomuceno et al., 2007). These genes might indicate “probiotic potential” by conferring to the strain a special ability to adhere to the intestinal epithelium. Besides, type I and III restriction- modification systems, iron homeostasis and plasmid replication genes could provide the strain with selective advantages over other coexisting bacteria.

**FIGURE 6:**
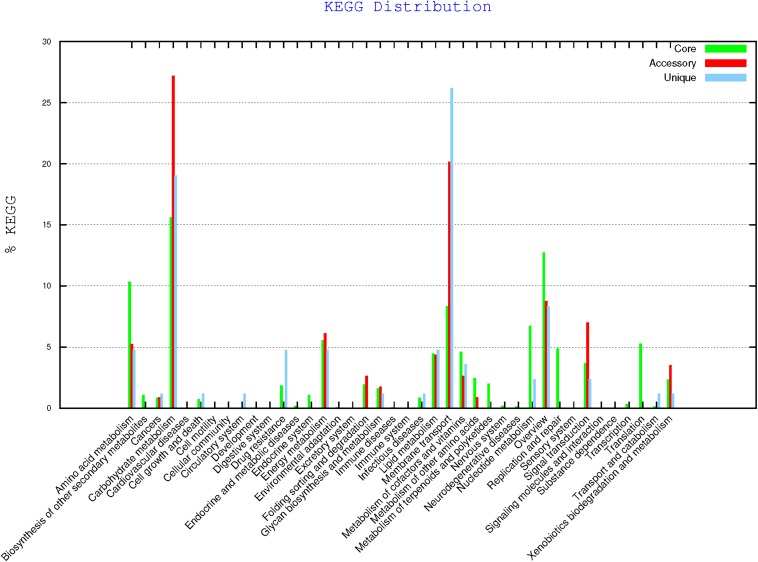
KEGG distribution of core, accessory and unique genes of *L. plantarum* strains (BPGA results).

Genomic comparison with BPGA program revealed the composition of the core genome of all strains resulting in 2,510 genes (Table 9), the accessory genes (existing in two or more strains), unique genes (exclusively present in the corresponding strain) and those exclusively absent in the corresponding strain (present in all genomes but not in the genome under study). Strain CECT 8962 has the highest number of unique genes among the five strains under study and also the highest number of exclusively absent genes. Distribution of genes belonging to core genome, accessory and unique, in KEGG families (Figure 6) supported that accessory and unique genes were mostly related to carbohydrate metabolism and membrane transport. On the other hand, genomic comparison confirmed that strains CECT 8963, CECT 8964, CECT 8965, and CECT 8966 were more similar among them than to CECT 8962 which could be explained by the fact that they were isolated from the same step in the fermentation process (M9, Elizaquível et al., 2015).

**TABLE 9:** Number of core, accessory, unique, and exclusively absent genes in *L. plantarum* strains calculated by BPGA program.

**Strain**	**No. of core genes**	**No. of accessory genes**	**No. of unique genes**	**No. of exclusively absent genes**
CECT 8962	2510	20	375	317
CECT 8965	2510	366	29	2
CECT 8963	2510	334	1	0
CECT 8964	2510	332	1	4
CECT 8966	2510	353	1	1

## Conclusion

This study details the workflow of the genome analysis carried out on five *L. plantarum* strains with biotechnological potential and highlights the usefulness of these tools to understand the singularity of strains as well as to assess their metabolic traits, technological abilities and safety concerns. Genomic analysis was carried out on strains CECT 8962, CECT 8963, CECT 8964, CECT 8965, and CECT 8966 previously isolated from *chicha* and biotechnologically characterized *in vitro*. Results from this study showed the five strains harbored the entire metabolic pathways of riboflavin and folates biosynthesis, supporting their corresponding phenotypic metabolic abilities. Food safety was also assessed following the EFSA recommendations, aiming at their food applications. Identification of all strains through genomic indexes confirmed that they belong to *L. plantarum* species which is included in the QPS list of EFSA. Regarding antibiotic resistance, the five strains encoded an aminoglycoside resistance gene, *aadA*, linked to streptomycin and spectinomycin resistance but such resistance was not detected *in vitro*. On the other hand, ampicillin resistance was observed previously *in vitro* in strains CECT 8964 and CECT 8966 but related genes were not found. No pathogenic factors were predicted for any of the strains. Four strains, with the exception of CECT 8962, harbored bacteriocin synthesis clusters for plantaricin E/F, A, and J. Regarding genome stability, prophage regions, plasmid genes and insertion sequences were present in addition to CRISPR and *cas* genes in the five strains, in accordance to the intrinsic genetic variability of the species *L. plantarum*. Finally, the genomic comparisons with RAST server tools and BPGA program, demonstrated that strain CECT 8962 encoded the major number of unique genes out of the five strains supporting its outstanding performance in previous studies (Yépez et al., 2019). This unique genome was composed of a large number of genes coding for proteins of unknown or hypothetical functions and also prophage and mobile elements. Many other exclusive genes were related to carbohydrates transport, modification, synthesis and degradation enzymes, as well as membrane proteins linked to adhesion to host cells. Overall, the genomic information warranties the safe use of these strains in food applications and opens new possibilities to exploit the biotechnological potential of singular strains.

## Data Availability Statement

The datasets generated for this study can be found in the OKQP01, OKQT01, OKQV01, OMOO01, and OMOP01.

## Author Contributions

RA and DA designed the study. LR-T obtained the genomes, did the mainstream processing, and drafted the manuscript. LR-T and AY analyzed the annotations. AY conducted the phenotypic testing supporting the genomic analysis. All authors corrected and approved the manuscript.

## Conflict of Interest

The authors declare that the research was conducted in the absence of any commercial or financial relationships that could be construed as a potential conflict of interest.

## Footnotes

1 https://www.kegg.jp/blastkoala/

2 https://www.genome.jp/kegg/genes.html

3 https://blast.ncbi.nlm.nih.gov/Blast.cgi

4 https://card.mcmaster.ca/analyze/rgi

5 https://cge.cbs.dtu.dk/services/PathogenFinder/

6 http://bagel4.molgenrug.nl/

7 http://phaster.ca/

8 https://crisprcas.i2bc.paris-saclay.fr/CrisprCasFinder/Index

9 https://isfinder.biotoul.fr

10 https://cge.cbs.dtu.dk/services/PlasmidFinder/

11 https://www.who.int/foodsafety/publications/antimicrobials-fifth/en/

12 http://www.cazy.org
